# Ion and Proton Transport In Aqueous/Nonaqueous Acidic Ionic Liquids for Fuel-Cell Applications—Insight from High-Pressure Dielectric Studies

**DOI:** 10.1021/acsami.1c06260

**Published:** 2021-06-24

**Authors:** Zaneta Wojnarowska, Alyna Lange, Andreas Taubert, Marian Paluch

**Affiliations:** † Institute of Physics, the University of Silesia in Katowice, Silesian Center for Education and Interdisciplinary Research, 75 Pulku Piechoty 1A, 41−500 Chorzow, Poland; ‡ Institute of Chemistry, University of Potsdam, Karl-Liebknecht-Straße 24-25, 14469 Potsdam-Golm, Germany

**Keywords:** proton hopping, dielectric spectroscopy, high pressure, ion transport, acidic ionic liquids

## Abstract

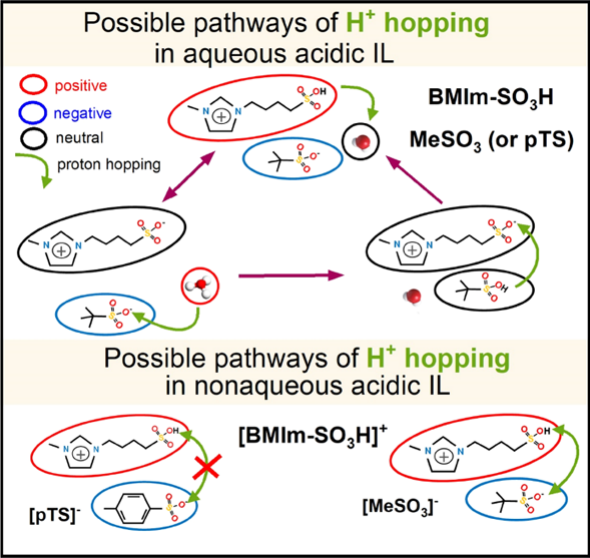

The use of acidic ionic liquids and solids as electrolytes in fuel cells is an emerging field due to their efficient proton conductivity and good thermal stability. Despite multiple reports describing conducting properties of acidic ILs, little is known on the charge-transport mechanism in the vicinity of liquid–glass transition and the structural factors governing the proton hopping. To address these issues, we studied two acidic imidazolium-based ILs with the same cation, however, different anions—bulk tosylate vs small methanesulfonate. High-pressure dielectric studies of anhydrous and water-saturated materials performed in the close vicinity of *T*_g_ have revealed significant differences in the charge-transport mechanism in these two systems being undetectable at ambient conditions. Thereby, we demonstrated the effect of molecular architecture on proton hopping, being crucial in the potential electrochemical applications of acidic ILs.

## Introduction

The fluids and solids made of ions and short-lived ion pairs have received considerable attention over the past 20 years.^1^ Due to practically unlimited combinations of cations and anions, they reveal multiple extraordinary physical properties, including excellent thermal^2^ and chemical stabilities, low vapor pressure,^3^ and nonflammability.^4^ These features make them attractive electrolytes for fuel cells that, in turn, are expected to become the most promising energy converters for automotive, stationary, and portable applications in the nearest future.^5^

To get an efficiently working fuel cell, an ionic electrolyte that enables fast proton transport is required. The simplest way to achieve this goal is electrolyte hydration since water shows a proton transfer within the Grotthuss mechanism, widely recognized as the quickest distribution of protonic defect.^6^ The critical issue in this strategy is a proper balance between water and other components; too much water drowns the cell, too little dehydrates it. Furthermore, water limits cell operation to a temperature range of 5–90 °C. Above the upper limit, water quickly evaporates, while under freezing conditions, it solidifies into ice. Both these processes substantially reduce proton mobility, leading to a decrease in dc conductivity, a drop in cell potential, and finally, a temporary power loss. Additionally, a volume increase during the water freezing can crush the cell. Therefore, much effort is currently taken to design fuel-cell electrolytes (FCEs) capable of proton transport under low-humidity conditions.^7,8^

In the absence of water, proton transport is realized in two ways: (i) in the so-called vehicle mechanism, i.e., together with a cation/anion, or (ii) by H^+^ hopping, being independent of ion diffusion.^9^ Since the former one is directly governed by structural relaxation (viscosity), an increase in proton mobility can be achieved only by accelerating ion transport, i.e., by designing electrolytes easily flowing at FC operating conditions.^10^ Nevertheless, this strategy can result in leakage from an electrochemical device that significantly limits commercial applications. Therefore, an approach involving fast proton transport strongly decoupled from structural rearrangements is a much more promising way to get highly conducting electrolytes.^11^

To make the proton transport independent of the bulk mechanical properties of an electrolyte, one needs to incorporate the functional groups, revealing a high ability to donate/accept H^+^ into a cation and/or an anion’s chemical structure. At least two proton-active sides and a well-organized H-bonding network are required to observe H^+^ conduction in a given material.^12^ This can be provided, e.g., by sulfonate and phosphate groups that, in analogy to water, form strong H bonds and participate in fast Grotthuss conduction.^6^ The molecules that undergo internal rearrangements of chemical bonds, like amide–imidic acid transformation, are also preferred.^13^ Nevertheless, the proton relays being a part of the chemical structure are not enough to observe efficient H^+^ hopping through the FCE. Molecular architecture is another critical factor. Theoretical models reveal that higher side-chain density and flexibility facilitate the mobility of protons in macromolecular systems.^14^ Interestingly, the covalent bonding of both cations and anions to the polymer chain in polymer blends has been found to bring the same effect.^15^ However, the bulk structure of some ions sterically blocks the proton hopping.^16^ Therefore, it still remains a challenge to recognize the set of molecular features supporting proton transfer and acting as a bottleneck. An additional difficulty is to design an electrolyte that reveals fast proton hopping in an FC operating window, i.e., from −20 to +80 °C. Since in every FCE, proton-transport mechanism depends on thermodynamic conditions and ion dynamics, the supercooling ability of electrolytes and the temperature of liquid–glass transition (*T*_g_) are of great importance. If the material is characterized by very low *T*_g_ (≈−90 °C) (e.g., for imidazolium-based PILs)^17^ around RT conditions, ions’ kinetic energy is high enough and packing density is low enough to harm the hydrogen bonds. Consequently, H^+^ hopping is suppressed, and the dc conductivity can be realized only by the mobility of ions.^18^ Despite that σ_dc_(RT) usually takes a satisfactorily high value, such an electrolyte is not suitable for commercial applications due to the poor mechanical properties, being a direct consequence of low *T*_g_. As a counterexample, in high *T*_g_ materials, having a structure of amorphous solid above RT, translational motions of ions are frozen at FC operating conditions, and H^+^ hopping becomes the only source of dc conductivity. In such a case, to get a sufficiently high value of dc conductivity, the proton transport needs to be strongly decoupled from ions’ structural rearrangements.^19^

According to the literature, acidic ionic liquids are characterized by a moderate value of *T*_g_, high conductivity, as well as good thermal, chemical, and electrochemical stabilities, and therefore can be successfully used as electrolytes for batteries and FC.^34^ These materials are defined as a low melting ionic salt with acidic characteristics provided by functional groups located in a cation, anion, or both. Especially interesting seem to be Brönsted acidic ionic liquids with H^+^ on acidic functional groups of the anion and the cation. This is due to the formation of strong H bonds and possible proton transport between ionic species. As an example, density functional theory studies of 1-(3-propylsulfonic)-3-methylimidazolium hydrogen sulfate [(HSO_3_)C_3_C_1_im][HSO_4_] show a great tendency to form strong intramolecular hydrogen bonds in a zwitterion; however, this tendency is weakened in the cation. In addition to the role of proton-transport pathways, the intra- and intermolecular hydrogen bonds that coexist in the ionic liquid are important in the stability of the systems.^20^ Interestingly, the dynamic simulations on −SO_3_H-functionalized ILs have also shown significant aggregations of sulfonic acid side chains due to strong interactions between different sulfonic acid groups.^21^ Successful utilization of acidic ionic liquids in functioning polymer electrolyte fuel cells has been already shown by Díaz^22^ and Skorikova^23^ who immobilized these imidazolium- and iminium cation-based ionic liquids in different matrices.

Interestingly, despite multiple reports on acidic ionic liquids available in the literature, little is known on the charge-transport mechanism in the vicinity of liquid–glass transition and the structural factors controlling the proton transport. A valuable insight into these issues can bring high-pressure experiments. It is well known that the density changes accompanying compression have a similar effect on ion mobility as isobaric cooling.^24^ However, an increase in the pressure under isothermal conditions affects only the packing density and conformational changes of ions while their kinetic energy (thermal energy) remains unchanged. Consequently, only by limiting the molecules’ free volume, one can reveal the structural factors affecting charge transport within the vehicle conduction and proton hopping.

The current paper focuses on studies of the charge-transport mechanism in two specifically designed acidic ionic liquids: 1-methyl-3-(3-sulfobutyl)-imidazolium *para*-toluenesulfonate (pTS), [BMIm-SO_3_H][pTS], and 1-methyl-3-(3-sulfobutyl)-imidazolium methanesulfonate, [BMIm-SO_3_H][MeSO_3_]. Due to the sulfonate/sulfonic acid group located in the chemical structure of ions as well as equimolar water concentration, both ILs should be capable of rapid proton transport of the same efficiency. Ambient-pressure dielectric measurements performed over a wide temperature range covering supercooled-liquid and glassy states have shown many similarities in the relaxation dynamics behavior of both studied systems. Therefore, to reveal the details of the charge-transport mechanism in these materials, high-pressure dielectric experiments have been employed. We show that isothermal compression provides a unique description of ionic motions in anhydrous and water-saturated ILs. Specifically, it explains the effect of molecular architecture on proton hopping, vehicle conduction efficiency, and the role of water in charge transport, all crucial in the potential electrochemical applications of acidic ILs.

## Experimental Section

### Synthesis of ILs

The ionic liquids were synthesized via a two-step process that is described in detail elsewhere.^25^ In short, in the first step, a zwitterion [BMIm-SO_3_] was synthesized by the reaction of 1-methylimidazol and 1,4-butane sultone in acetone. The resulting white powder was then used in the second step to obtain the ionic liquids by mixing the zwitterion with the respective acid (methanesulfonic acid or *p*-toluenesulfonic acid) in equimolar amounts.

Elemental analysis for BMIm-SO_3_ (C_8_H_14_N_2_O_3_S; *M* = 218.27 g/mol) calculated (found): C, 44.0 (43.8); H, 6.5 (6.5); N, 12.8 (12.8); S, 14.7 (15.1). ^1^H NMR (600 MHz, D_2_O): δ [ppm]: 8.72 (s, 1H), 7.48 (s, 1H), 7.42 (s, 1H), 4.23 (t, 2H), 3.87 (s, 1H), 2.92 (t, 2H), 2.25–1.81 (m, 2H), 1.83–1.31 (m, 2H).

Elemental analysis for [BMIm-SO_3_H][pTS] (C_15_H_22_N_2_O_6_S_2_; *M* = 390.09 g/mol) calculated (found): C, 46.1 (44.9); H, 5.7 (6.1); N, 7.2 (7.0); S, 16.4 (15.6). ^1^H NMR (300 MHz, D_2_O): δ [ppm]: 8.60 (s, 1H), 7.60 (d, *J* = 8.3 Hz, 2H), 7.30 (dd, *J* = 22.4, 12.6 Hz, 4H), 4.11 (t, *J* = 7.1 Hz, 2H), 3.77 (s, 3H), 2.85 (t, 2H), 2.30 (s, 3H), 2.08–1.75 (m, 2H), 1.75–1.48 (m, 2H).

Elemental analysis for [BMIm-SO_3_H][MeSO_3_] (C_9_H_18_N_2_O_6_S_2_; *M* = 314.37 g/mol) calculated (found): C, 34.4 (33.1); H, 5.8 (6.6); N, 8.9 (8.6); S, 20.4 (19.5). ^1^H NMR (300 MHz, D_2_O): δ [ppm]: 8.64 (s, 1H), 7.38 (d, 2H), 4.16 (t, *J* = 7.0 Hz, 2H), 3.80 (s, 3H), 2.85 (t, 2H), 2.71 (s, 3H), 2.14–1.81 (m, 2H), 1.81–1.47 (m, *J* = 23.3, 7.7 Hz, 2H).

### Differential Scanning Calorimetry (DSC)

Calorimetric experiments of the studied compounds were performed using a Mettler Toledo DSC1STAR System equipped with a liquid nitrogen cooling accessory and an HSS8 ceramic sensor (a heat flux sensor with 120 thermocouples). Temperature and enthalpy calibrations were performed using indium and zinc standards.

### Broadband Dielectric Spectroscopy (BDS) at Ambient and Elevated Pressure

Ambient-pressure dielectric measurements of ILs were performed over a wide frequency range from 10^–2^ to 10^6^ Hz using a Novo-Control GMBH Alpha dielectric spectrometer. The same sample was used for the ambient- and high-pressure measurements (capacitor of diameter 10 mm, distance 0.1 mm). However, for high-pressure experiments, the sample was additionally protected by Teflon tape. The dielectric spectra were collected over a wide temperature and pressure range. The room temperature was chosen as a high-temperature limit to avoid water evaporation during the experiment. For ambient-pressure measurements, the temperature was controlled by a Novo-Control Quattro system with the use of a nitrogen gas cryostat. On the other hand, a Weise fridge was used to control the temperature in high-pressure experiments. In both cases, the temperature stability was equal to 0.2 K.

### Mechanical Measurements

The mechanical measurements were performed using an ARES G2 Rheometer. Aluminum parallel plates with a diameter of 8 mm were used during the experiments. The rheological experiments were performed in the frequency range from 0.1 to 100 rad/s (10 points per decade) with the strain equal to 0.01% in the vicinity of the liquid–glass transition. The strain was increased by 1 order of magnitude with every 10 K.

### Thermal Analysis

Simultaneous thermogravimetric analysis–differential thermal analysis (TGA–DTA) experiments were done on a Linseis L81 thermal balance and on a Linseis STA PT-1600 thermal balance in the air from 20 to 900 °C with a heating rate of 10 K/min.

## Results and Discussion

### Sample Characterization

**Scheme 1:**
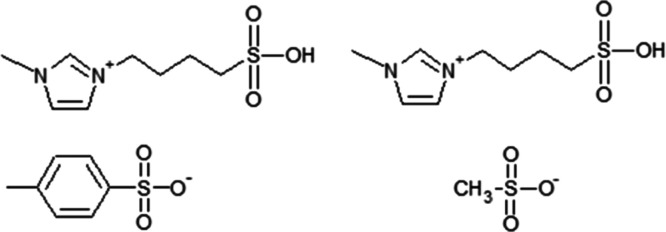
Chemical Structures of Studied ILs: [BMIm-SO_3_H][pTS] (on the Left) and [BMIm-SO_3_H][MeSO_3_] (on the Right)

The materials examined herein were chosen as model systems to understand the effect of molecular architecture on the charge-transport mechanism in acidic ionic liquids. Specifically, a butyl-imidazolium cation terminated by a sulfonate group (BMIm-SO_3_H) was chosen to create two salts with *para*-toluenesulfonate (pTS) and methanesulfonate (MS) anions, respectively (see Scheme 1). As a consequence, both ILs are characterized by a very similar capability to donate/accept H^+^ and have the potential to favor proton hopping in anhydrous conditions. However, due to the rigid toluene in the anion’s architecture of [BMIm-SO_3_H][pTS], the charge-transport mechanism can be different in these two ILs. Additionally, it is expected to change dramatically in the presence of water. Specifically, the acidic SO_3_H groups, located on the side chains of the imidazolium cation, dissociate into SO_3_^–^ and free protons when exposed to water. Since the latter can be readily accepted by water molecules or R-SO_3_^–^ anions, the hydrated ILs become a mixture of imidazolium-based zwitterions, BMIm-SO_3_H cations, H_3_O^+^, R-SO_3_^–^ anions, and neutral R-HSO_3_ molecules. From this perspective, the charge transport can be realized by ions, protons, and protonated water molecules.

**Figure 1:**
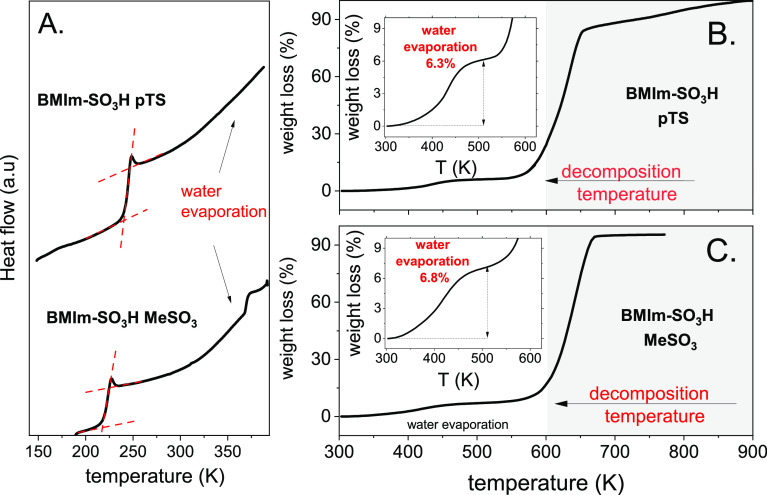
(A). DSC thermograms of studied acidic ILs. (B) and (C) TGA scans of analyzed samples.

The differential scanning calorimetry and TGA method were employed to provide an initial description of the studied materials. The DSC traces obtained during the heating of ILs are presented in Figure 1A. In addition to the heat flow jumps, being a typical feature of the liquid–glass transition, each DSC thermogram contains a broad endotherm related to water evaporation.

Since this effect is comparable for studied ILs, a similar water content is expected in both these materials. The exact water fraction was determined using TGA experiments. As shown in Figure 1B,C, in the temperature range from 300 to 510 K, there is a mass loss of 6.8 wt % for [BMIm-SO_3_H][MeSO_3_] and 6.3 wt % for IL with a pTS anion. These values recalculated to a mole fraction of water (*x*_H_2_O_) are equal to 0.55 and 0.58, respectively, which means approximately one molecule of water per one ion pair. To ensure that the observed mass loss was correctly ascribed to water evaporation, the new set of samples was heated up to 510 K and then tested using the NMR technique. The obtained NMR data did not reveal any signs of decomposition and confirmed the excellent thermal stability of studied ILs. Therefore, in the next sections, we focus on studying the charge-transport mechanism in these systems.

### Dielectric Studies of Hydrated ILs at Ambient Pressure

**Figure 2:**
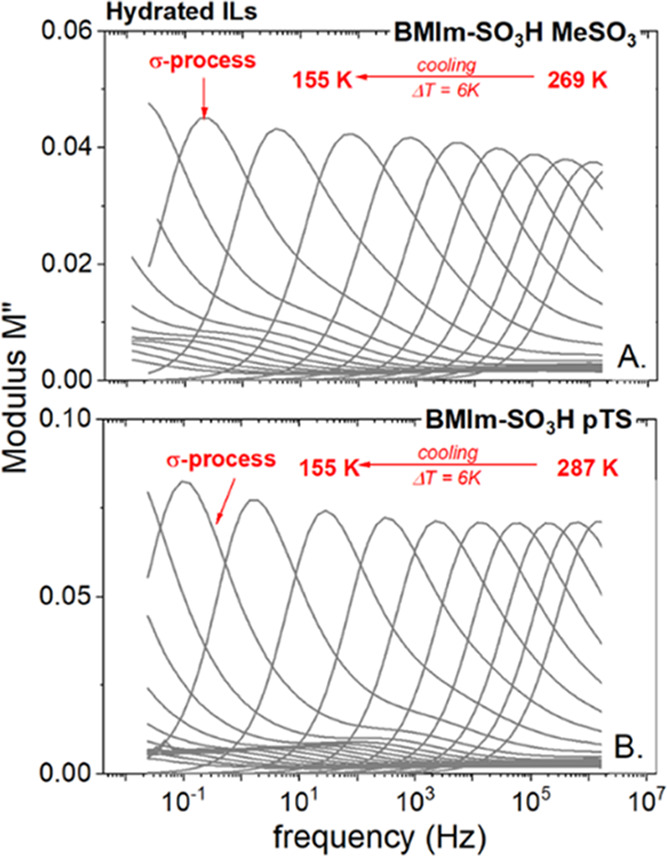
Representative dielectric data of hydrated samples: [BMIm-SO_3_H][MeSO_3_] (A) and [BMIm-SO_3_H][pTS] (B), recorded at ambient-pressure conditions.

The dielectric measurements on hydrated [BMIm-SO_3_H][MeSO_3_] and [BMIm-SO_3_H][pTs], having *x*_H_2_O_ equal to 0.55 and 0.58, respectively, were performed over a broad range of frequency from 10^–2^ to 10^6^ Hz. According to the literature, there are several approaches frequently used to analyze the dielectric response of an ionic material, including complex impedance Z*(*f*), permittivity ε*(*f*), modulus M*(*f*), and conductivity σ*(*f*) formalisms.^26^ However, among mentioned, the modulus representation M*(*f*) = *M*′ + i*M*″ is the most convenient for thorough characterization of ion motions, and thereby identifying the charge-transport mechanism in a given material.^27^ Therefore, we have used this formalism for further analysis of dielectric data collected for acidic ILs. The representative *M*″(*f*) spectra of [BMIm-SO_3_H]-based ILs are depicted in Figure 2. As can be seen, in both examined cases, the imaginary part of the modulus function *M*″(*f*) takes the form of a well-resolved peak denoted as a σ-process and ascribed to the translational motions of ionic species. Additionally, with decreasing temperature, the *M*″ loss peak shifts toward lower frequencies. This behavior is directly connected with a significant decrease in ion mobility accompanying cooling and is quasiuniversal for all kinds of ionic glass formers, including classical ionic liquids,^28^ protic ILs,^29^ and even ion-conducting polymers.^30^

Therefore, to provide a detailed description of charge transport in studied systems, the temperature evolution of the *M*″ loss peak has to be examined in more detail.

**Figure 3:**
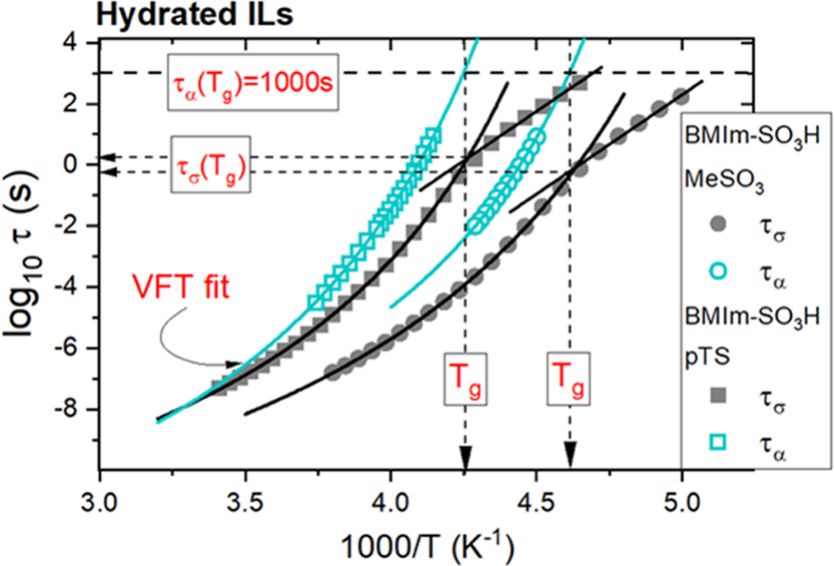
Temperature dependence of conductivity relaxation times and structural relaxation times determined for hydrated ILs. Solid lines denote the fit of the VFT equation τ_σ_ = τ_σ_0__exp(D*T*_0_/(*T* – *T*_0_)) to the experimental data. The straight lines denote fits of Arrhenius low to the experimental points.

The temperature evolution of conductivity relaxation times (τ_σ_ = ε_0_ε_s_/σ_dc_) determined directly from the *M*″ peak maximum (τ_σ_ = 1/2πf_max_) is presented in Figure 3. The log_10_τ_σ_(1000/T) data of both examined acidic ILs exhibit qualitatively similar features, i.e., follow a non-Arrhenius behavior over a broad T-range and reveal a well-defined crossover from the Vogel–Fulcher–Tamman (VFT) to Arrhenius dependence at a specific temperature (*T*_cross_). Typically *T*_cross_ remains in good agreement with the value of calorimetric liquid–glass transition (*T*_g_),^31^ and this is also the case of examined ILs. However, in both analyzed samples, τ_σ_(*T*_cross_) is in the order of 1 s instead of 10^3^ s, commonly identified with the freezing point of ion motions at *T*_g_.^11^ Thus, τ_σ_(*T*_g_) < 10^3^ s indicates that the charge transport still occurs in hydrated ILs when the ions are already immobilized in the glassy state. This, in turn, is the first sign that [BMIm-SO_3_H]-based ILs reveal fast proton conductivity, being to some extent independent of cation and anion motions and additionally independent of H_3_O^+^ diffusion. A direct comparison between the time scale of conductivity relaxation and structural dynamics (τ_α_) is needed to verify this hypothesis. For aprotic materials with vehicle-type conduction, the time scale of structural dynamics and conductivity relaxation are the same at different T–P conditions. This is because the charge can be transported only when ions move. Then, the common practice is to say that these two quantities are coupled. On the other hand, there are two contributions to charge transport for protic ionic systems: translational diffusion of ions (having the same time scale as structural relaxation) and proton hopping, independent of ionic species’ motions. Due to the additional contribution of proton conductivity, the time scale of conductivity relaxation is different (faster) than the time scale of structural relaxation. The second scenario is expected for, herein, studied acidic ionic liquids.

**Figure 4:**
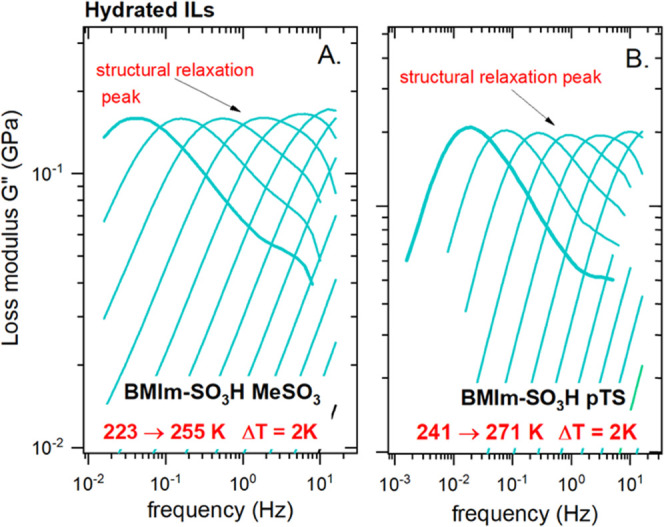
Modulus loss data *G*″(*f*) of [BMIm-SO_3_H][MeSO_3_] (A) and [BMIm-SO_3_H][pTS] (B) measured by means of an ARES G2 rheometer.

A common practice to determine structural relaxation time, τ_α_, of ion-containing systems is to employ mechanical spectroscopy. The representative results of mechanical measurements performed for acidic ILs are depicted in Figure 4.

As one can see, the frequency dependence of shear loss modulus *G*″ forms a well-visible peak, just like the electric modulus. However, this time, the peak maximum corresponds to the structural relaxation time τ_α_ = 1/2πf_max_. Note that the structural relaxation reflects the time scale of translational motions of all of the species existing in a given system (both ionic and neutral). Since the *G*″(*f*) maxima can be easily identified only in the vicinity of the liquid–glass transition, the time-temperature superposition (TTS) principle was employed to probe the mechanical relaxation in a supercooled-liquid state. From the relaxation map presented in Figure 3, it becomes evident that at any considered temperature, the time scale of structural rearrangement is longer than that corresponding to charge transport, and this difference becomes larger with decreasing temperature. Furthermore, τ_α_(*T*_g_) estimated from the VFT fitting curve’s extrapolation is found to be equal to 10^3^ s, which is typical for all glass formers. However, at the same time, it is much longer than the time scale of conductivity relaxation at *T*_g_. Thus, the charge transport in hydrated acidic ILs is indeed partially independent of structural dynamics. A similar picture, i.e., so-called decoupling between τ_α_ and τ_σ_, has been observed in the past for multiple proton-conducting materials.^32,33^ As a consequence, the origin of the time scale separation between τ_α_ and τ_σ_ in studied ILs could be automatically ascribed to fast proton transport. Nevertheless, such a mechanism is not the only one that might decouple charge transport from mass diffusion. From studies of single-ion conductors, it is well known that an alternative reason is the different contributions of cations and anions to ionic conductivity and structural relaxation.^34^ To unambiguously verify which scenario is true for studied hydrated acidic ILs, we took advantage of high-pressure dielectric measurements. Recently, this method has been presented as a powerful tool for understanding the conductivity mechanism in ionic glass formers.^16^ Specifically, it has been demonstrated that the decoupling phenomenon becomes more significant at elevated pressure for proton-conducting systems. On the other hand, the opposite behavior is a feature of decoupled aprotic ionic polymers for which the free volume is a decisive factor governing the charge transport.

### Dielectric Studies of Hydrated ILs at Elevated Pressure

**Figure 5:**
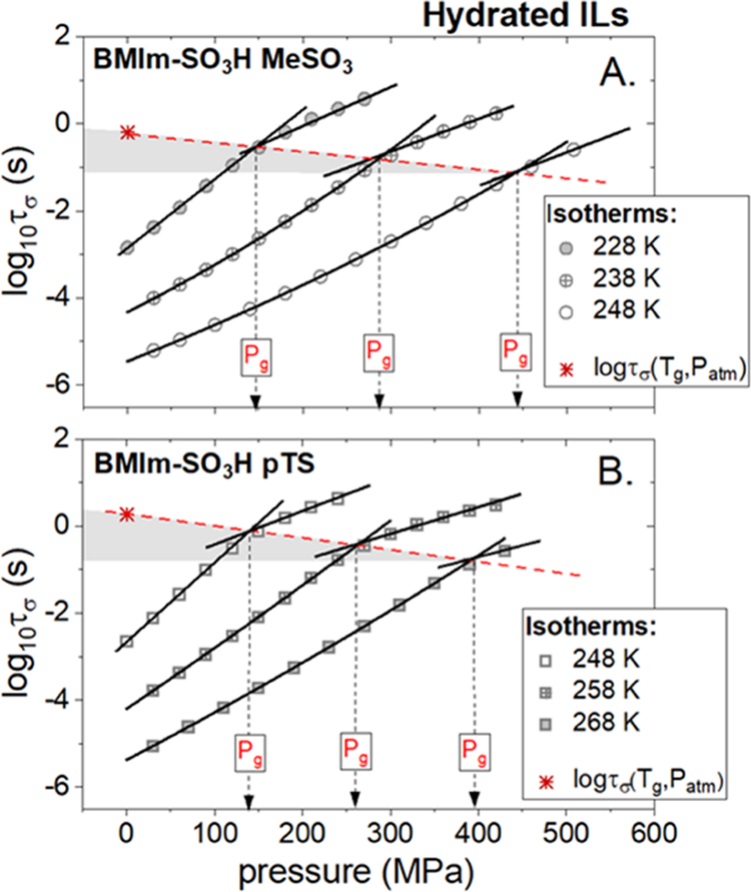
Pressure dependences of conductivity relaxation times measured at different isothermal conditions for hydrated ILs: [BMIm-SO_3_H][MeSO_3_] (A) and [BMIm-SO_3_H][pTS] (B). The experimental data recorded at *P* < *P*_g_ were parameterized by means of the pressure counterpart of the VFT_p_ equation: τ_σ_ = τ_σ_0__exp(CP/(*P* – *P*_0_)).

According to the standard experimental protocol,^35^ the examined ILs were compressed isothermally at three different temperatures, each higher than *T*_g_. The obtained log τ_σ_(*P*) dependencies are illustrated in Figure 5A,B for [BMIm-SO_3_H][MeSO_3_] and [BMImSO_3_H][pTS], respectively.

Since the isothermal compression has the same effect on ion dynamics as isobaric cooling, the τ_σ_ of supercooled ILs gets longer with pressure. Additionally, in analogy to ambient-pressure conditions, logτ_σ_ markedly slows down when the liquid–glass transition is transgressed. Consequently, the crossover of log τ_σ_(*P*) again appears; however, this time, at the glass-transition pressure (*P*_g_). From closer inspection of Figure 5, it is also visible that the value of conductivity relaxation time at *P*_cross_ becomes shorter when the compression at a higher temperature is performed. This fact, together with a report demonstrating that the kink of log τ_σ_ always occurs at isochronal structural relaxation time (τ_α_ ≈ 10^3^ s)^36^ both at ambient and elevated pressure, indicating that compression enhances the decoupling between τ_σ_ and τ_α_ in the investigated ILs. In other words, the charge transport becomes more efficient under conditions of high compression. This result confirms the Grotthuss mechanism’s contribution to overall charge transport in examined hydrated [BMImSO_3_H]-based ILs. Of course, it does not mean that the proton hopping is the only source of dc conductivity in studied supercooled systems (it is the only source of charge transport in the glassy state). The time scale of conductivity relaxation covers the diffusion of ions: anions, protonated water, cations (to less extend), and, additionally, the proton hopping between ions/molecules. Neutral molecules, i.e., zwitterions, water molecules, and R-HSO_3_ do not contribute to charge transport. However, it should be noted that due to the intensive proton hopping, there is a dynamic equilibrium between ionic and neutral species. In this context, it is interesting to ask which of the studied ILs reveals more efficient proton transport?

### Charge Transport in [BMIm-SO_3_H][MeSO_3_] and [BMIm-SO_3_H][pTS]—A Comparison

**Figure 6:**
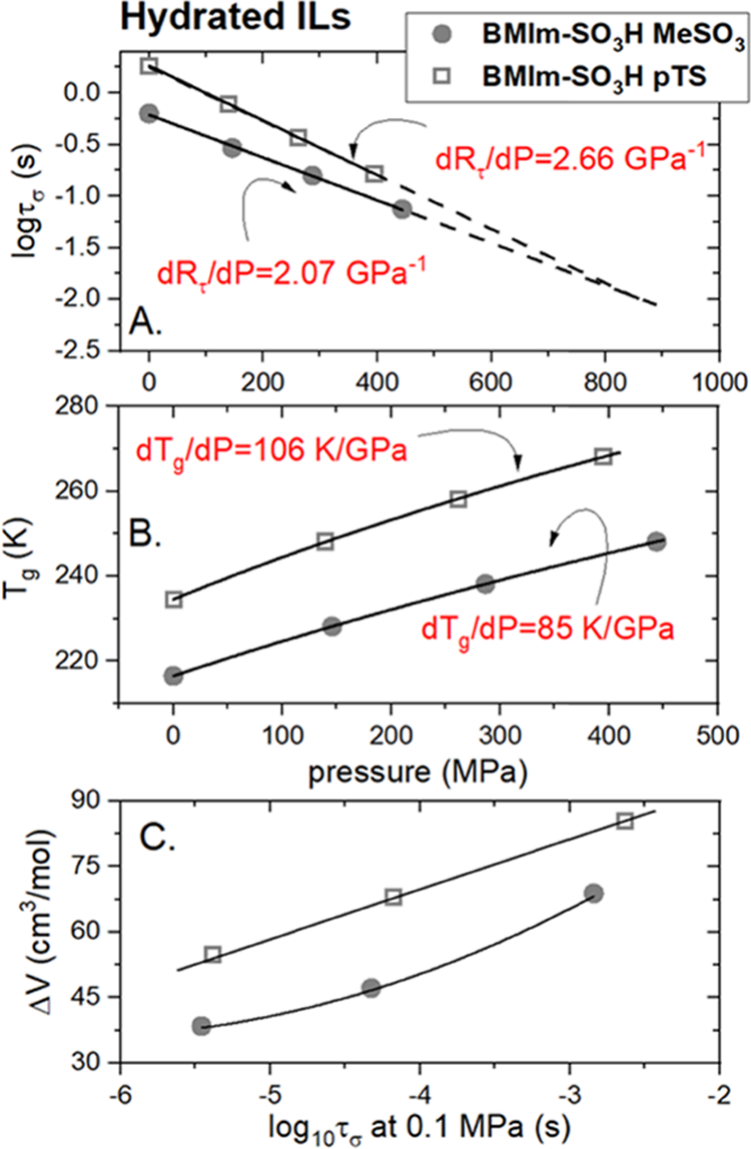
(A) Pressure behavior of log τ_σ_(*T*_g_) determined for hydrated ILs. Dashed lines present the extrapolation of linear fits. (B) Pressure behavior of *T*_g_ for hydrated ILs. (C) Apparent activation volume is determined in the limit of ambient pressure and plotted as a function of log τ_σ_ at 0.1 MPa.

To shed light on this problem, one can take advantage of the decoupling parameter *R*_τ_(*T*_g_) and its pressure behavior. According to literature reports, *R*_τ_(*T*_g_) is defined as the difference between the time scale of structural dynamics and conductivity relaxation at the liquid–glass transition, i.e., *R*_τ_(*T*_g_) = 3 log τ_σ_(*T*_g_).^37^ Therefore, it is frequently considered a quantitative measure of a decoupling phenomenon and the process staying behind, namely, H^+^ transport in proton-conducting systems. Applying this rule, hydrated [BMIm-SO_3_H][MeSO_3_] with *R*_τ_(*T*_g_) = 3.20 reveals slightly stronger decoupling than water-saturated [BMIm-SO_3_H][pTS] (*R*_τ_(*T*_g_) = 2.75), and therefore can be considered as a better proton conductor. The same is true also in high-pressure conditions. Namely, in the experimentally available pressure range (0.1–450 MPa), *R*_τ_(*T*_g_) determined for [MeSO_3_]-based IL is always higher than that of [BMIm-SO_3_H][pTS] (see Figure 6A). Another interesting observation is the pressure sensitivity of the log τ_σ_(*T*_g_) expressed by the d*R*_τ_/d*P* parameter. Namely, hydrated IL with a pTS anion reveals steeper log τ_σ_(*T*_g_)(P) dependence than the MeSO_3_-based sample, which is manifested by a higher d*R*_τ_/d*P* coefficient equal to 2.66 GPa^–1^. Thus, considering these two examined ILs, isothermal compression affects proton transport more in [BMIm-SO_3_H][pTS]. To identify the molecular origin of this observation, one needs to look closer at the chemical structure of studied ILs. Due to the butyl-imidazolium cations terminated by a SO_3_H group, anions with a sulfonate group and water molecules, all existing in an equimolar concentration in studied ILs, the number of moieties actively involved in proton transport, as well as the proton donor–acceptor capability, are the same for both materials. The only structural difference between examined ILs lies in the type of substituent covalently attached to the negatively charged SO_3_ group of the anion, namely, bulky toluene vs a small CH_3_ group in [BMIm-SO_3_H][pTS] and [BMIm-SO_3_H][MeSO_3_], respectively. Since the former one sterically blocks the H-bond formation, the number of “highways” for fast proton hopping is limited in the pTS-based compound. This brings a lower value of *R*_τ_(*T*_g_) in this system. On the other hand, when the free volume is markedly reduced in the compressed material and the [BMIm-SO_3_H] cations and pTS anions get closer to each other, new H bonds can be formed. This makes H hopping more efficient. At the same time, the H-bonded network in [BMIm-SO_3_H][MeSO_3_], well-organized already at 0.1 MPa, is less sensitive to density changes. Interestingly, to get the same conducting properties of both acidic ILs (the same log τ_σ_(*T*_g_)), squeezing up to 0.9 GPa is required (see Figure 6A). At this pressure condition, the intermolecular distance is expected to be the same in both samples.

### Insight into the H-Bonding Network from High-Pressure Studies

In the context of the discussion presented above, the following question arises: whether or not the H-bonding interactions in [BMIm-SO_3_H][MeSO_3_] are indeed stronger than in [BMIm-SO_3_H][pTS]? To address this issue, one can exploit the fact that the glass-forming liquids characterized by a well-expanded H-bonding network reveal weaker pressure sensitivity of molecular dynamics when compared to simple van der Waals liquids.^38^ This rule is also fulfilled for various ionic systems, including protic ionic liquids or ionic polymers.^39,16^ Among the parameters quantifying the effect of pressure on relaxation dynamics in ionic glass formers, one can mention: (i) apparent activation volume^40^ defined as Δ*V*^#^ = 2.303RT(d log τ_σ_/d*P*)_T_ and (ii) the d*T*_g_/d*P* coefficient describing how much *T*_g_ is sensitive to pressure variations. Directly from the Δ*V*^#^ definition, it is clear that the higher the Δ*V*^#^ value, the greater is the change in τ_σ_ upon compression, and thereby higher pressure sensitivity of given IL. A similar trend is also followed by the d*T*_g_/d*P* coefficient. Herein, it is essential to note that the apparent activation volume also reflects the size of relaxing units. Namely, the smaller the ions contributing to charge transport, the lower is ΔV^#^. As a consequence, H^+^ migration should also decrease the apparent activation volume.

**Figure 7:**
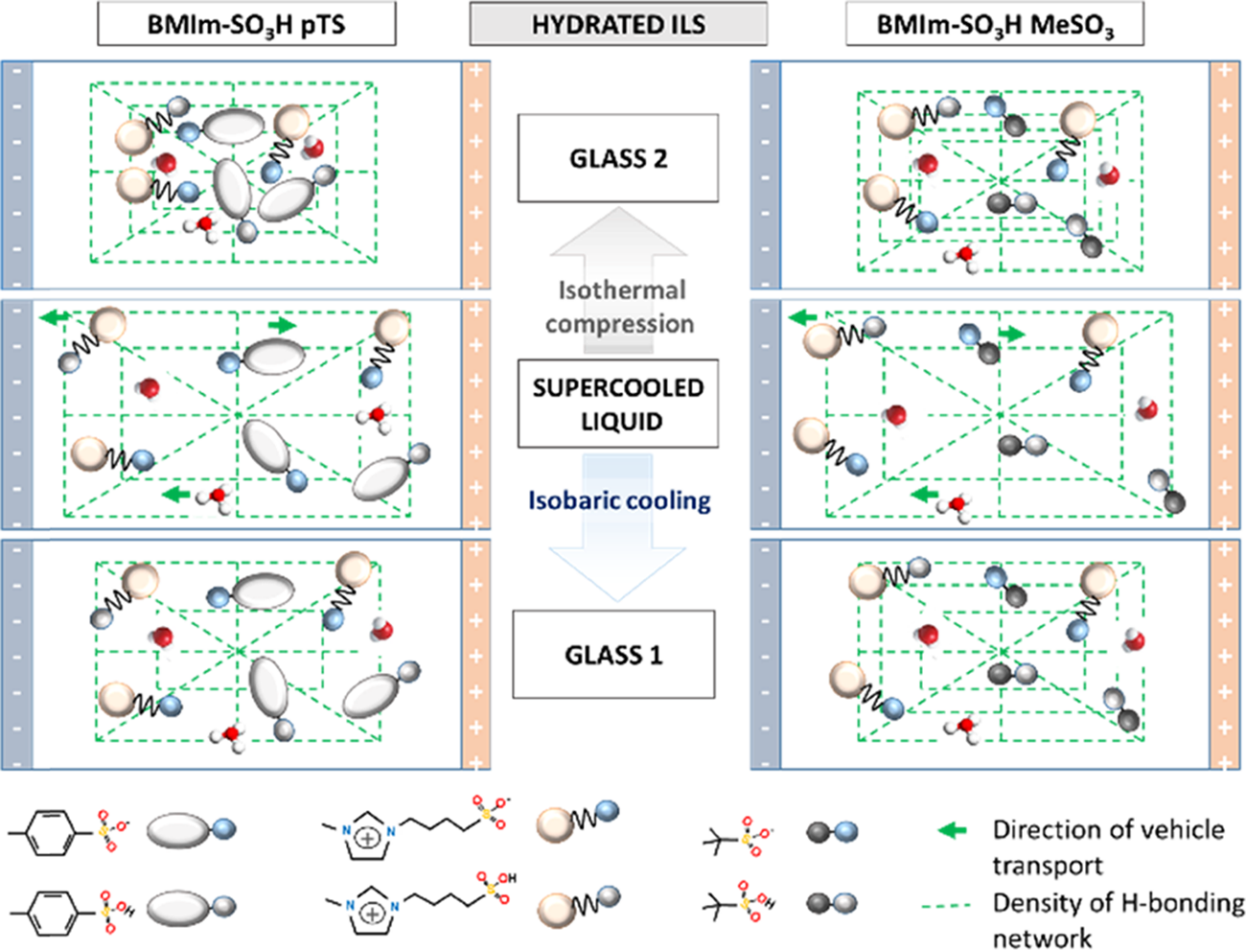
Visualization of H bonding and charge transport in hydrated ILs. The ion transport in supercooled-liquid and two glassy states (obtained during isobaric cooling and isothermal compression) is presented. In the glassy state, the mobility of the ion becomes frozen and H^+^ hopping through the H-bonding network is the only source of charge transport. The efficiency of proton transport in the compressed glass is higher due to higher density (around six times) compared to the material obtained during the vitrification process. The larger pressure sensitivity of [BMIm-SO_3_H][pTS] is also visualized. It is also shown that the supercooled ILs contain mostly neutral zwitterions, anions, and protonated water; however, neutral H_2_O and R-HSO_3_ molecules and cations can also exist in the system.

Δ*V*^#^ for the same initial mobility of ions (the same τ_σ_) needs to be calculated to compare the pressure sensitivity of different compounds. This is due to the strong temperature dependence of the activation volume, generally observed for glass-forming systems.^36^ Additionally, due to the nonlinear character of τ_σ_(*P*) dependencies, the value of Δ*V*^#^ is usually calculated in the limit of ambient pressure.^41^ Δ*V*^#^ data coming from the present experiments are shown in Figure 6C. As can be seen, at any examined τ_σ_, Δ*V*^#^ determined for [BMIm-SO_3_H][MeSO_3_] is smaller than Δ*V*^#^ for the pTS-based compound, and the difference becomes relatively constant with an increase in ion mobility. This indicates that (i) smaller ions contribute more to charge transport in MeSO_3_-based IL (i.e., MeSO_3_^–^, H^+^, H_3_O^+^) while larger ions contribute to charge transport in pTS-IL (e.g., pTS^–^) and (ii) the structure of [BMIm-SO_3_H][MeSO_3_] creates more highways (H bonds) for H^+^ transport. The same conclusion can be drawn from the analysis of the d*T*_g_/d*P* coefficient that is lower for IL with a MeSO_3_ anion. To visualize the density of the H-bonding network and possible pathways of proton migration in examined acidic ILs, the structure of the studied material is schematically illustrated in Figure 7.

In light of the presented results, it is evident that the charge transport in examined ILs is dominated by protons released during the dissociation of HSO_3_ groups located in the cation. Consequently, the examined systems are in fact mixtures of ions (anions, H_3_O^+^, and a small number of cations) and neutral molecules (mostly zwitterions; however, also some R-SO_3_H and H_2_O) that are actively involved in proton hopping. Thus, the efficiency of H^+^ hopping is expected to decrease significantly when the amount of water becomes limited. To shed more light on this issue in the next part of this paper, we will focus on the ion dynamics of [BMIm-SO_3_H][MeSO_3_] and [BMIm-SO_3_H][pTS] containing a reduced amount of water.

### Effect of Water on the Temperature of Liquid–Glass Transition in Studied Acidic ILs

**Figure 8:**
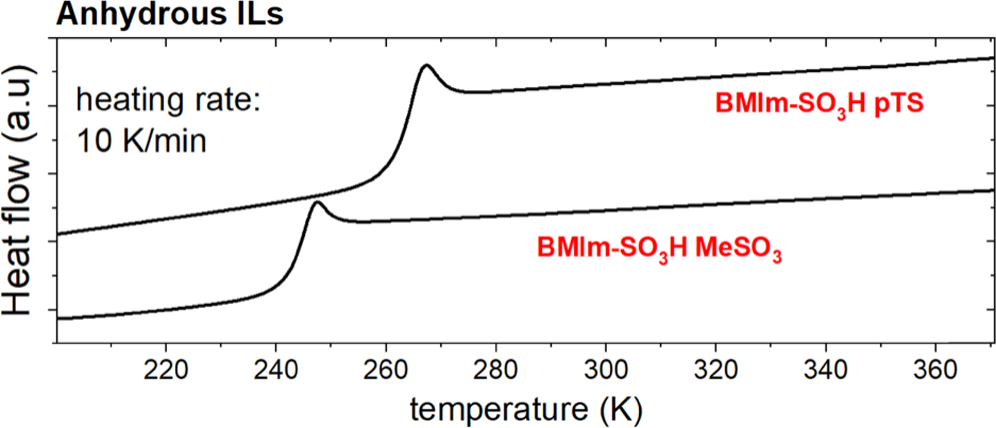
DSC traces of anhydrous ILs.

It is well known that even a small amount of water absorbed by any type of low molecular liquid affects its molecular dynamics. Usually, it plasticizes the structure that is manifested by a decrease in glass-transition temperature. This effect can be large or small, depending on the hygroscopicity of a given compound. For instance, in the case of a 3PG–water mixture, there was only an 8 K difference between the anhydrous sample and the mixture containing *x*_H_2_O_ = 0.83.^41^ On the other hand, an increase in the water content in lidocaine hydrochloride from zero to *x*_H_2_O_ = 0.44 causes a drop of *T*_g_ by about 42 K (which is almost 15% of the value determined for anhydrous sample).^42^ However, the most significant effect of water on *T*_g_ was observed for protic polymerized ionic liquid poly-[HSO_3_-BVIm][OTf], where *T*_g_ was increased by 80 K with a decrease in the water content from 11 to 1 wt %. Due to the acidic nature of ILs examined herein, the prominent effect of water on *T*_g_ is expected for these systems. To explore this issue, the DSC measurements of [BMIm-SO_3_H]-based ILs were performed. In the beginning, the starting materials with the highest water content (*x*_H_2_O_ = 0.55 and 0.58) were annealed at 393 K for 15 min. Next, the samples were cooled to 200 K (20 K/min), and the subsequent dynamic calorimetric measurements with a heating rate of 10 K/min were performed. Such a procedure was repeated multiple times until the tested samples’ mass and *T*_g_ became constant. The final thermograms are depicted in Figure 8. As can be seen, apart from the characteristic signature of *T*_g_, there is no trace of water in studied materials. Additionally, in both examined acidic ILs, there is a 20° growth in *T*_g_.

### Effect of Water on the Decoupling Phenomenon and the Charge-Transport Mechanism in Studied ILs

The thin film of the sample was dried directly on a capacitor plate under vacuum at *T* = 373 K for 48 h to address this issue. Such a procedure enabled us to obtain anhydrous [BMIm-SO_3_H][MeSO_3_], while the pTS-based compound still contained 0.5% of water, as determined by Karl-Fisher titration. In the further part of this paper, both these materials will be denoted as dried.

**Figure 9:**
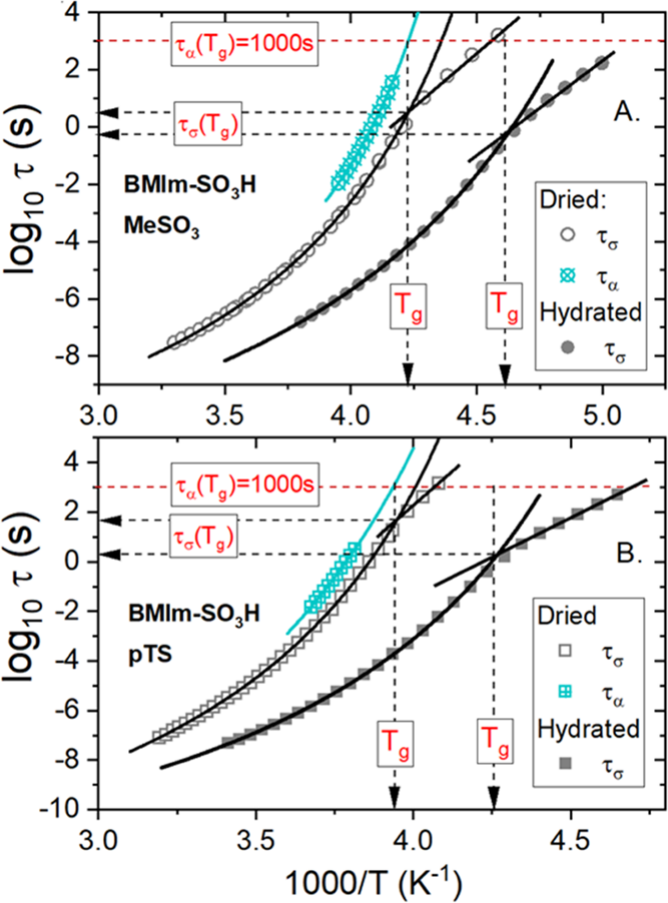
Temperature dependence of conductivity relaxation times for dried and hydrated ILs: [BMIm-SO_3_H][MeSO_3_] (A) and [BMIm-SO_3_H][pTS] (B). For dried samples, the temperature behavior of structural relaxation is also presented.

First, we explored the effect of water on the decoupling phenomenon in acidic ILs. For this purpose, dielectric spectroscopy in combination with mechanical measurements was again employed. Since the loss modulus *M*″(*f*) and *G*″(*f*) spectra reveal quite universal features, these data are not shown. On the other hand, the temperature dependences of conductivity and structural relaxation times obtained directly from modulus peaks maxima are presented in Figure 9.

Like highly hydrated materials, the cooling of dry samples results in a dramatic increase of τ_σ_. Additionally, in the whole examined temperature range, τ_σ_ of dried compounds is longer than the time scale of conductivity relaxation reported above for water-saturated samples. Moreover, the kink of τ_σ_(*T*^–1^) dependence from VFT-like to Arrhenius behavior still occurs, however, at a longer conductivity relaxation time. The difference in τ_σ_(*T*_g_) between hydrated and dried materials reaches 1.5 decades for [BMIm-SO_3_H][pTS] and less than 1 decade for [BMIm-SO_3_H][MeSO_3_]. On the other hand, τ_α_(*T*_g_) maintains a constant value of 1000 s. As a consequence, the decoupling between charge transport and structural dynamics still occurs, but it is smaller in water-free materials. It means that the charge transport is still faster than the structural dynamics. Consequently, the fast proton hopping still contributes to conducting properties of the dried ILs, however, to much less extent than it was for water-saturated compounds. The role of Grotthuss conduction in overall charge diffusion is especially reduced in dried [BMIm-SO_3_H][pTS]. Since *R*_τ_(*T*_g_) dropped down to 1.1, the vehicle mechanism seems to be dominant in this IL. In other words, dc conductivity comes mainly from the diffusion of cations and anions. Additionally, due to the small amount of water still remaining in this sample (0.5%), one can assume that the charge transport would be fully coupled to structural dynamics in an anhydrous material. In other words, pure [BMIm-SO_3_H][pTS] itself cannot be classified as a proton-conducting liquid. A quite different situation takes place for [BMIm-SO_3_H][MeSO_3_]. Even a water-free material is characterized by *R*_τ_(*T*_g_) = 2.5 at ambient-pressure conditions (Figure 9). This means that the water contribution is not obligatory to observe the Grotthuss conduction in this system.

### Pressure Sensitivity of Ion Dynamics in Dried [BMIm-SO_3_H]-Based ILs

**Figure 10:**
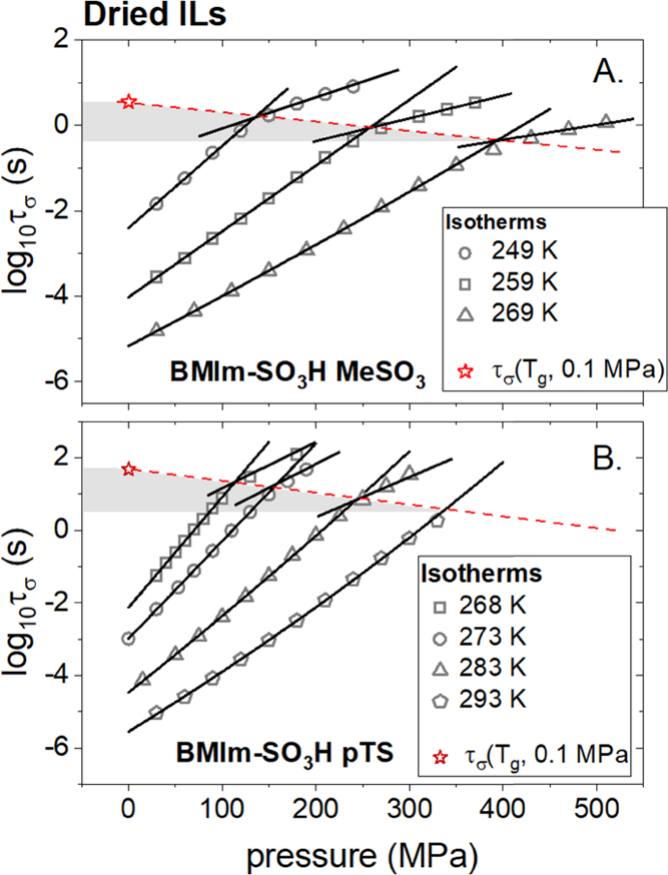
Pressure dependences of conductivity relaxation times measured at different isothermal conditions for dried ILs: [BMIm-SO_3_H][MeSO_3_] (A) and [BMIm-SO_3_H][pTS] (B). The experimental data recorded at *P* < *P*_g_ were parameterized by means of the pressure counterpart of the VFT_p_ equation: τ_σ_ = τ_σ_0__exp(CP/(*P* – *P*_0_)).

The isothermal dielectric measurements in the pressure range of 0.1–500 MPa have been performed to advance this problem. Herein, it is important to note that we used the same samples and the same capacitor for high-pressure experiments as it was for ambient-pressure measurements. The obtained set of isothermal curves are presented in Figure 10A,B for dried [BMIm-SO_3_H][MeSO_3_] and [BMIm-SO_3_H][pTS], respectively.

As observed previously for water-saturated samples, log τ_σ_(*P*) dependences recorded at *P* < *P*_g_ reveal a non-Arrhenius character well parameterized by the pressure counterpart of the VFT equation. On the other hand, above *P*_g_, the Arrhenius behavior is observed. Another similarity is the shortening of log τ_σ_(*P*_g_) with an increasing temperature that indicates some participation of proton hopping in overall charge transport, as concluded above. Nevertheless, this contribution is markedly smaller in comparison to water-saturated systems, especially for IL with pTS anion. Interestingly, the pressure behavior of the decoupling phenomenon in water-free [BMIm-SO_3_H][MeSO_3_] is practically the same as *R*_τ_(*P*) found for highly saturated [BMIm-SO_3_H][pTS]. The further analysis of crossover points indicates that *T*_g_(*P*) dependencies are also the same for these two systems. Hence, the strength of the H-bonding network seems to be equally strong in both these ILs. Consequently, some H^+^ donors or acceptors in [BMIm-SO_3_H][pTS] are most likely deactivated. This is probably due to the bulky structure of a tosylate anion that sterically blocks the H-bonding formation and proton transport. This hypothesis finds confirmation in X-ray diffraction (XRD) and Fourier transform infrared (FTIR) studies of [PMIm-SO_3_H][pTS], IL having the same structure as [BMIm-SO_3_H][pTS] with the only difference in the length of the alkyl substituent (propyl instead of butyl).^43^ Namely, it has been found that the SO_3_ groups on the side chain of the imidazolium cation share a proton with a SO_3_ group attached on the side chain of a neighboring imidazolium cation. The same behavior is observed on the SO_3_ group of the IL anion. Additionally, alternating layers of the IL cations with an overall positive charge and a tosylate anion with an overall negative charge have been detected. Consequently, in water-free [PMIm-SO_3_H][pTS], the proton’s movement from the cation to anion is hampered. Therefore, water serves as a proton mediator between oppositely charged species.

**Figure 11:**
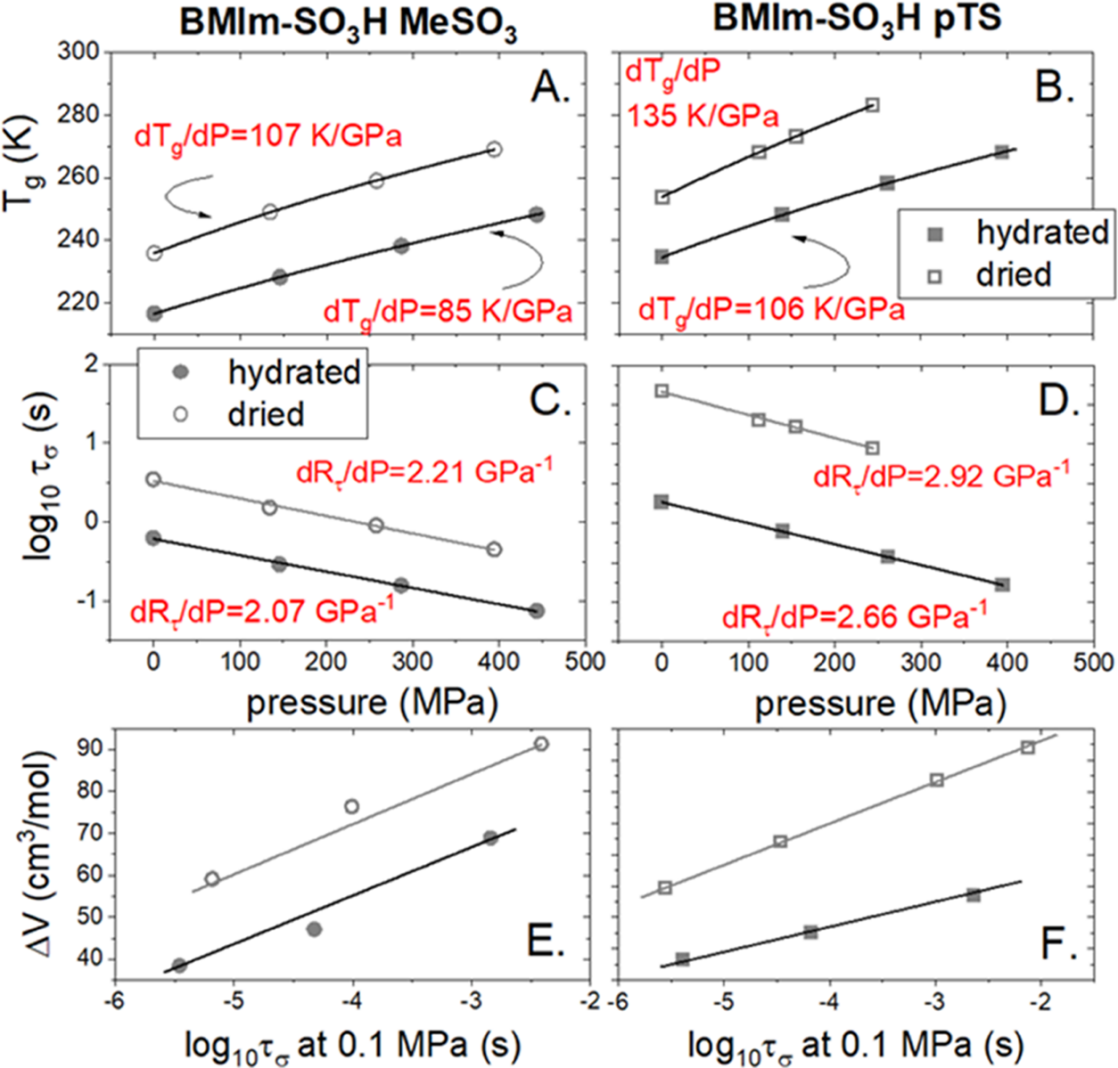
(A, B) Pressure dependence of *T*_g_ for studied ILs. (C, D) Pressure behavior of log τ_σ_ determined at the liquid–glass transition. (E, F) Behavior of apparent activation volume is presented as a function of log τ_σ_. Open symbols: dried materials and closed symbols: hydrated samples.

**Figure 12:**
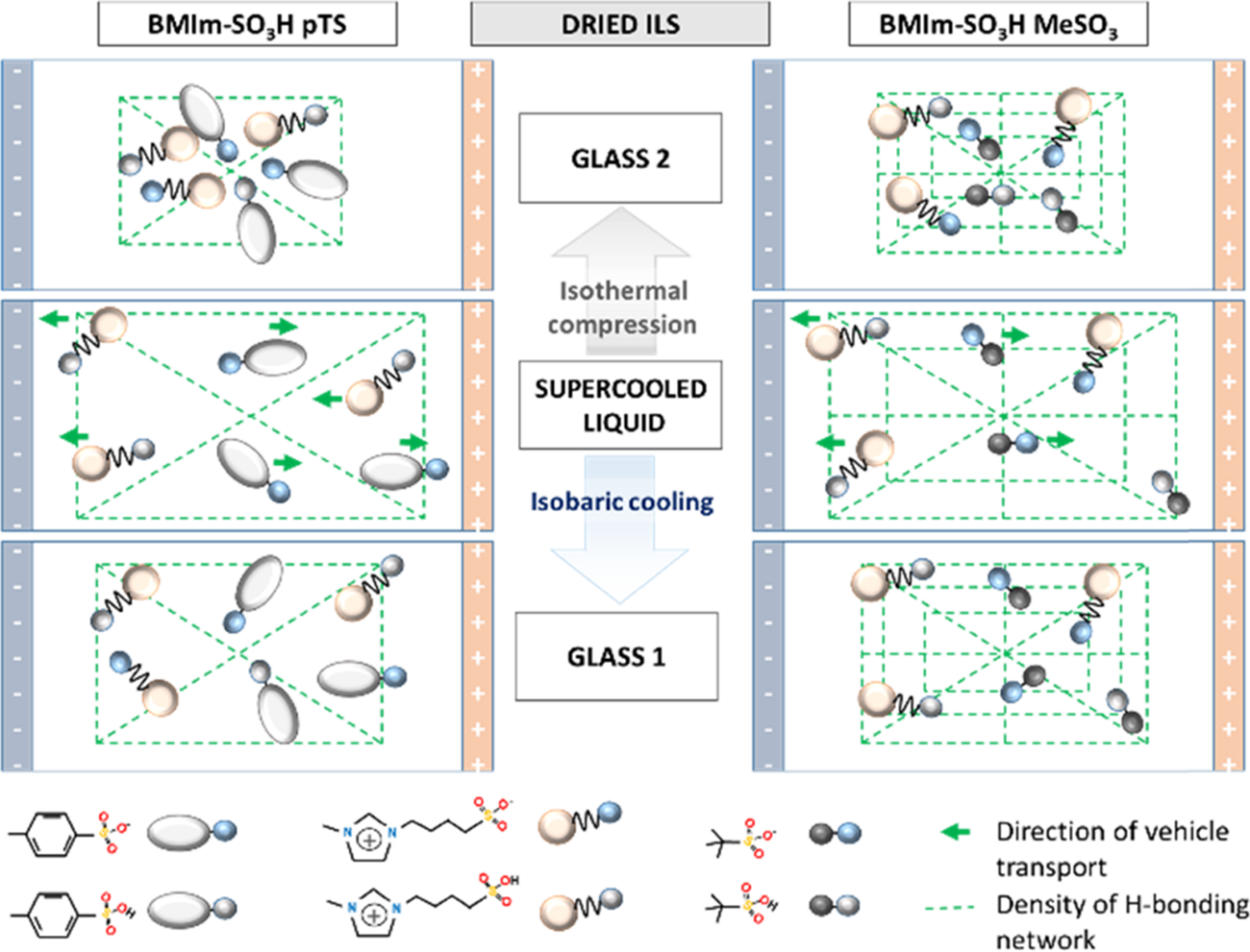
Visualization of charge transport and density of the H-bonding network in dried acidic ILs. In comparison with hydrated samples (Figure 7), the density of the H-bonding network is much smaller in dried materials; however, more distinct changes are observed between hydrated and dried [BMIm-SO_3_H][pTS] anions. Due to the lack of water, the number of imidazolium zwitterions existing in the supercooled-liquid state of [BMIm-SO_3_H][MeSO_3_] is strongly limited. At the same time, the supercooled state of dry [BMIm-SO_3_H][pTS] contains only cations and anions. Zwitterions appear in the compressed material due to the proton hopping between the cation and the anion.

When the high-pressure dynamics of hydrated and anhydrous IL is further compared, one can see that the *T*_g_(*P*) dependence is steeper in dried materials (see Figure 11A,B). This is quantified by a higher value of the d*T*_g_/d*P* coefficient. Specifically, for [BMIm-SO_3_H][MeSO_3_], an increase from 85 to 107 K/GPa is observed, while the change from 106 to 135 K/GPa is denoted for [BMIm-SO_3_H][pTS]. The higher pressure sensitivity of ion dynamics in dried ILs is also reflected in the behavior of the apparent activation volume. As presented in Figure 11E,F, Δ*V*^#^ increases with a decrease in the water content for both studied materials; however, the change is much more pronounced in [BMIm-SO_3_H][pTS]. Namely, Δ*V*^#^ increases by about 70% with a decrease in the water content. This result again indicates that the H-bonding network in hydrated [BMIm-SO_3_H][pTS] is created mostly by water molecules. Consequently, water seems to be the main molecule mediating the Grotthuss transport in hydrated pTS-based IL. On the other hand, the quite weak pressure sensitivity of ion dynamics in both hydrated and anhydrous [BMIm-SO_3_H][MeSO_3_] indicates that the H-bonding network does not change much after water evaporation, and in both cases, is good enough to work as an efficient highway for proton conduction. Consequently, among the studied acidic ILs, anhydrous [BMIm-SO_3_H][MeSO_3_] can be considered a potential electrolyte for fuel cells. The visualization of H-bonding network density and the charge-transport mechanism in dried acidic ILs is presented schematically in Figure 12.

## Conclusions

In this paper, we investigated the ion dynamics in two imidazolium-based ionic liquids of the same cation [BMIm-SO_3_H] and different anions, [MeSO_3_] vs [pTs], in supercooled liquid and glassy states. We found that both hydrated (*x*_H_2_O_ ≈ 0.5) and dried materials can be classified as good glass-forming liquids without any crystallization tendency. The temperature of liquid–glass transition markedly increases with drying (Δ*T* = 20 K) and reaches 243 and 262 K for anhydrous [BMIm-SO_3_H][MeSO_3_] and [BMIm-SO_3_H][pTS], respectively. Ambient-pressure dielectric measurements combined with rheological experiments have shown that both samples reveal decoupling between charge transport and mass diffusion when containing an equimolar amount of water. This indicates a contribution of proton hopping to the overall charge-transport mechanism in studied water-saturated ILs. Note that the Grotthuss conduction is the most efficient in the close vicinity of liquid–glass transition, i.e., when the vehicle transport becomes frozen. Since the decoupling index *R*_τ_ determined at *T*_g_ differs only by 0.5 between these ILs, one can conclude that molecular architecture of anions does not affect the proton transport efficiency much in the examined systems. The high-pressure experiments, providing a substantial increase in the system density (around six times larger than it is during isobaric cooling), confirm the role of Grotthuss conduction in charge transport, however, at the same time highlight the differences between properties of hydrated [BMIm-SO_3_H][MeSO_3_] and [BMIm-SO_3_H][pTS]. Namely, (i) a better organized H-bonding network in IL with a methanesulfonate anion, i.e., less sensitive to pressure changes and having more “highways” for H^+^ hopping and (ii) a larger distance between H-donor/acceptor moieties in pTS-based IL. Notably, ambient- and high-pressure dielectric studies of dried systems revealed that water molecules play a crucial role in proton transport of [BMIm-SO_3_H][pTS], while they are not critical to observe fast H^+^ hopping in [BMIm-SO_3_H][MeSO_3_]. Specifically, due to an aromatic ring located in the tosylate anion, the H^+^ exchange between cations and anions is blocked in the anhydrous material, and the charge transport becomes dominated by simple vehicle conduction. The pronounced pressure sensitivity of dried [BMIm-SO_3_H][pTS] also indicates poor construction of the H-bonding network in this system. On the other hand, [BMIm-SO_3_H][MeSO_3_] can be classified as a good proton conductor both under high-humidity and water-free conditions.
